# IBES: a tool for creating instructions based on event segmentation

**DOI:** 10.3389/fpsyg.2013.00994

**Published:** 2013-12-26

**Authors:** Katharina Mura, Nils Petersen, Markus Huff, Tandra Ghose

**Affiliations:** ^1^German Research Center for Artificial Intelligence (DFKI)Kaiserslautern, Germany; ^2^Department of Psychology, University of TübingenTübingen, Germany; ^3^Department of Psychology, University of KaiserslauternKaiserslautern, Germany

**Keywords:** event perception, instructions, assembly tasks, multimedia

## Abstract

Receiving informative, well-structured, and well-designed instructions supports performance and memory in assembly tasks. We describe IBES, a tool with which users can quickly and easily create multimedia, step-by-step instructions by segmenting a video of a task into segments. In a validation study we demonstrate that the step-by-step structure of the visual instructions created by the tool corresponds to the natural event boundaries, which are assessed by event segmentation and are known to play an important role in memory processes. In one part of the study, 20 participants created instructions based on videos of two different scenarios by using the proposed tool. In the other part of the study, 10 and 12 participants respectively segmented videos of the same scenarios yielding event boundaries for coarse and fine events. We found that the visual steps chosen by the participants for creating the instruction manual had corresponding events in the event segmentation. The number of instructional steps was a compromise between the number of fine and coarse events. Our interpretation of results is that the tool picks up on natural human event perception processes of segmenting an ongoing activity into events and enables the convenient transfer into meaningful multimedia instructions for assembly tasks. We discuss the practical application of IBES, for example, creating manuals for differing expertise levels, and give suggestions for research on user-oriented instructional design based on this tool.

Well-designed assembly instructions that ease the process of setting up or building new products are critical for improving user experience (Daniel and Tversky, 2012). For example, we appreciate if we can immediately make sense of a manual that tells us how to assemble a new furniture or gadget. In industrial work, instruction manuals support workers in the final assembly of different product variants accompanied by many complex manual actions and a lot of tools and components. Again, well-designed assembly instructions are a prerequisite for successful and competitive production.

Designing good instruction manuals is hard. A good assembly instruction consists of an optimum usage of textual and pictorial information and appropriately guides attention to especially important or difficult operations while de-emphasizing unnecessary details. The creation of good instruction manuals requires time and effort in becoming acquainted with the task, structuring its content, editing appropriate descriptions, finding and incorporating additional, clear pictorial information, and so on. Unfortunately, instruction manuals are often developed very late in the design process and under high time pressure shortly before new product variants are introduced (Gorecky et al., 2013). Thus, the ability to produce high-quality instructions, which incorporate textual and visual information in a way comprehensible to the user, is a key requirement to better usability of modern products and better productivity in industrial manufacture.

More specifically, instruction manuals should be based on cognitive principles in order to ensure improved processing and understanding of the task. First, there is a rich body of cognitive science literature confirming a multimedia effect on instructions. It suggests that people follow instructions and remember the involved actions more efficiently when instruction manuals incorporate multimodal information (i.e., graphical and textual information; Paivio, 1986). Second, research on event perception (Zacks and Tversky, 2003) suggests that the temporal structure of the instruction manual (i.e., the sequence of the instructional steps) should be based on natural perceptual processing. If these prerequisites are given, instructions will facilitate and advance perception, understanding, and memory of a task.

In this paper, we describe Instructions Based on Event Segmentation (IBES), a software tool that produces a ready-to-use multimedia instruction manual based on a video of the assembly task in question. The IBES tool requires users to segment the visual information within a stream of video frames into meaningful segments with start and end points. Yet, it is an empirical question if the structure of offline and deliberately created instructions corresponds to the natural perception of a task. We compare the instruction manuals produced using the IBES tool with online natural event segmentation. For that, we examine the step-by-step boundaries of the manuals and the event boundaries originating from natural event perception in an event segmentation task. The study shows that, even if the process of segmentation within the IBES tool is different from online natural event segmentation, the instructions created by the IBES tool have event boundaries corresponding to those from natural event perception.

IBES is the first software tool that supports the production of manuals that incorporate multimedia and are meaningfully structured. Our initial validation and experiences with IBES suggest that it is a simple, user-friendly, and automated way to produce high-quality instruction manuals.

## Literature review

Assembly instructions are “messages that guide people to perform procedural tasks by describing the steps or rules required for completing the task” (after Eiriksdottir and Catrambone, 2011, p. 750). Good assembly instructions convey the necessary information in a step-by-step manner (Heiser et al., 2004) and contain an appropriate amount of textual and pictorial information (Martin and Smith-Jackson, 2005). Here, we propose a tool for creating step-by-step assembly instructions from any video.

The IBES tool provides an option to combine textual labels with the visual information created by the tool. The dual coding theory (Paivio, 1986) suggests that verbal and pictorial information are processed independently in working memory and implies that pictures together with textual descriptions are superior in communication of information compared to pictures or descriptions alone. This hypothesis has been shown to hold for procedural instructions in several studies (e.g., Zacks and Tversky, 2003; Mayer et al., 2005) and was further elaborated in the Cognitive Theory of Multimedia Learning (Mayer, 2005). The benefits of graphics in combination with text for instructions and learning have been labeled as the *multimedia effect*. Previous studies even showed that this effect is especially prevalent in procedural tasks (Van Genuchten et al., 2012) where presentations of combined media reduces the load on working memory by supporting the processing of the actual task (Zacks and Tversky, 2003; Brunyé et al., 2006). Furthermore, the combination of both graphical and verbal media enables the optimal balance of their respective advantages and disadvantages (Horz and Schnotz, 2008). For instance, while a text is presented in a linear order, a picture more easily displays spatial relationships and parallel actions. On the other hand, text is more precise in contrast to pictures which often benefit from additional verbal guidance (Horz and Schnotz, 2008; Huff and Schwan, 2012).

The IBES tool follows the above mentioned design requirement for instructions that gives the generated manual a meaningful and comprehensible structure. For procedural tasks a step-by-step structure is most efficient (Agrawala et al., 2003; Daniel and Tversky, 2012). This is in line with predictions from theories on dynamic event perception. For example, the event segmentation theory (Zacks and Tversky, 2001; Kurby and Zacks, 2007) states that observers make sense of an ongoing activity by segmenting the stream of information in meaningful, hierarchically structured events. Between two events participants perceive event boundaries. Event boundaries can be assessed by the event segmentation method (Newtson and Engquist, 1976; Zacks et al., 2001): participants are instructed to indicate when they perceive that one meaningful event has ended and the next meaningful event begins. Research has shown that memory for these event boundaries is better compared to non-event boundaries (Newtson et al., 1977) and that the segmentation into meaningful events corresponds with the temporal regulation of attention (Huff et al., 2012). Further, filmic summaries of a task like cleaning a pistol that were edited such that they included the event boundaries were recalled more coherently than filmic summaries that did not include these event boundaries (Schwan and Garsoffky, 2004). Instructions designed according to the event structure yielded a better performance than unstructured instructions (Zacks and Tversky, 2003). Similarly, pauses at the point of event boundaries in instructional videos have a positive effect on cognitive load (Spanjers et al., 2010; Van Gog et al., 2010), whereas pauses between boundaries have more negative consequences on performance (Adamczyk and Bailey, 2004).

Thus, literature supports that multimedia instructions with textual and graphical information structured according to natural event boundaries would enhance the perception and understanding of procedural tasks. Until now, there is no method of creating such high quality instruction manuals easily and directly from a video of the procedural tasks. IBES is the first software tool that supports the design of multimedia instructions based on the natural human event perception processes.

## Overview of IBES tool

The IBES tool is released as freeware and is available at http://www.ict-cognito.org/demo. It is based on the approach for automatic task segmentation and instructions generation, described in Petersen and Stricker (2012). The users control the tool via a conventional point-and-click interaction, i.e., by using a desktop and a mouse.

### Technical background

The IBES tool runs on the Windows 64-bit platform. The graphical front-end is written using QML, a declarative markup language for writing graphical user interfaces which is part of Qt Framework. Some additions have been made to the original code for steps, such as, reading and writing text files. These were implemented in C++ and included through the QML plugin mechanism.

The formatted paper manuals are being generated in HTML using jQuery. For each of the steps two, three, and four (see below), the software writes a corresponding HTML document into the results folder. These documents and the “results.csv” file are saved and get updated on every user input, e.g., for printing intermediate results.

Qt is licensed under a LGPL license and jQuery under the MIT license. The tool is freely available and we request the users to cite the associated publications when using the tool in scientific publications.

### User interaction

Before starting the instruction creation with the IBES tool, the video being used for instruction creation, has to be transferred into an image sequence of “.jpg” files with names consisting of 5-digit number beginning from “00000.jpg,” “00001.jpg,” “00002.jpg,” and so on. The steps involved in creation of the image sequence are as follows. First, Virtual Dub, a free open source software (Lee, 2010) can be used to transform video files to image sequences via a simple command in the drop-down menu: “File” → “Export” → “Image sequence.” In the following dialog box one has to leave the field for filename prefix empty but enter “.jpg” as a filename suffix (without quotation marks) and set the minimum number of digits in name to 5. Then, one specifies the directory to hold the image sequence. It is important to remember the name and location of this directory as it will be required later. Finally, one has to specify the output format to JPG.

**Figure 1:**
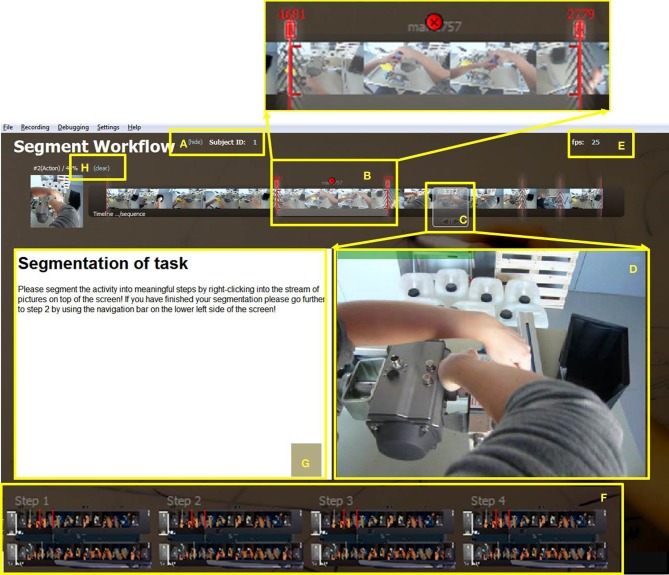
**Screenshot of start screen where segmentation into appropriate instructional steps takes place.** The subject ID **(A)** may be entered and hidden here. **(B)** Start and end frames of a step are indicated by two red marks which may be used to drag the boundaries (additionally amplified on top of this figure). The chosen step can be deleted by clicking the red circle in the middle. The white window **(C)** highlights the current picture which appears enlarged in the center of the screen **(D)**. The default frame rate value of 25 fps may be changed **(E)**. The navigation bar **(F)** at the bottom of the screen enables flexible moving back and forth between the four steps of the IBES tool, i.e., segmentation (step 1), choice of video frames (step 2), adding texts (step 3), and print preview (step 4). A field for instructions or information for the user is available **(G)**. The complete segmentation may be deleted by clicking on “clear” **(H)**.

Once the image sequence has been created, it has to be copy-pasted from the chosen directory into the “sequence” folder of the IBES tool. Afterwards, by activating the file “create new sequence.bat” the images are imported automatically and the actual IBES workflow can be started by clicking “start.bat.” The start screen (representing step 1) contains the complete stream of frames of the video appearing as a filmstrip on top of the screen (see Figure 1).

As can be seen from Figure 1E, a default value of 25 frames per second (fps) is assumed in the tool. Virtual dub (Lee, 2010) can be used to get the frame rate of the video (“Video” → “Frame Rate”). If it is different from this value, the user or experimenter may change it by editing the text directly on the first screen.

The subject ID is situated on the left side (see Figure 1A) and is “1” by default. In order to change it, e.g., in a user study involving different subjects, it can be replaced by typing the actual subject ID directly into the field. One can remove the subject ID from the screen by clicking “hide” so that it cannot be changed during this session.

Additionally, there is a navigation bar containing four buttons at the bottom of the screen (Figure 1F). The four buttons enable moving forward and backward within the four steps of the IBES tool. The first step is choosing segments from the video frames representing instructional steps. The second step is to select the most important and most representative frames for each instructional step. For the third step, subjects see their preliminary manual consisting of instructional steps and pictures. They can add textual descriptions for each instructional step. In the fourth step, the completed manual is shown to the user. It can be printed and manually added by visual overlays (e.g., arrows). In the following paragraphs, we describe each step of the instruction creation process.

On the start screen, the users receive the task to segment the sequence of video frames into instructional steps (see Figure 1G). More specifically, the users choose segments from the stream of frames by mouse clicks. The chosen pictures and the corresponding frame numbers are shown in a small, transparent window (Figure 1C) and additionally amplified in a bigger window below the stream (Figure 1D). If the users hold down the left mouse key while moving over the stream of pictures, the big window shows a movie clip consisting of the marked pictures.

Specifically, for segmenting the stream of pictures the white transparent window in Figure 1C has to be placed at the starting point of a new step followed by a right mouse click. A default time window (see Figure 1B) with two red marks appears when the mouse is moved a little above the filmstrip. Then the users adjust the end point by dragging the right red boundary to the appropriate end frame. The two red boundary-marks represent the start and end frames of an instructional step that should go into the instruction manual. The subsequent instructional step for the manual can start with the very next frame after the end frame of the preceding segment. However, if the immediate next frames are not meaningful, the start point can be moved forward until the next important step begins. The users may delete a step by clicking on the cross displayed above the selection window (see Figure 1B). By pressing “Clear” on the upper left side of the screen (see Figure 1H) they may delete the entire segmentation.

**Figure 2:**
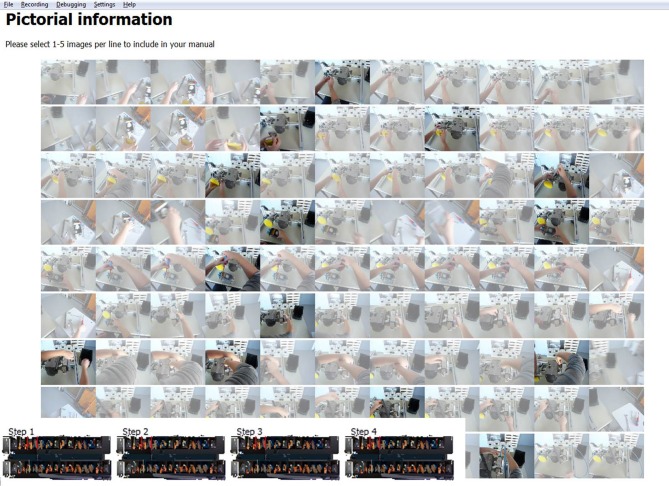
**Screenshot for step 2 within the IBES tool where users choose appropriate pictures representing each instructional step.** Pictures that have been chosen for the manual are shown more clearly than the rest.

After the segmentation is complete, the users have to navigate to the second step using the navigation bar (Figure 1F). As shown in Figure 2, each of the event segments chosen in step 1 of the IBES tool workflow appear in a separate row on a new screen (in Figure 2 nine steps are displayed for clarity). By default, each of the event segments is displayed as a sequence of eleven images. The users' task is to choose the essential and most representative pictures that are to be incorporated into the instruction manual by clicking on them. The users usually choose at least one picture from every event segment. Users may cancel their selection by clicking on the picture again.

**Figure 3:**
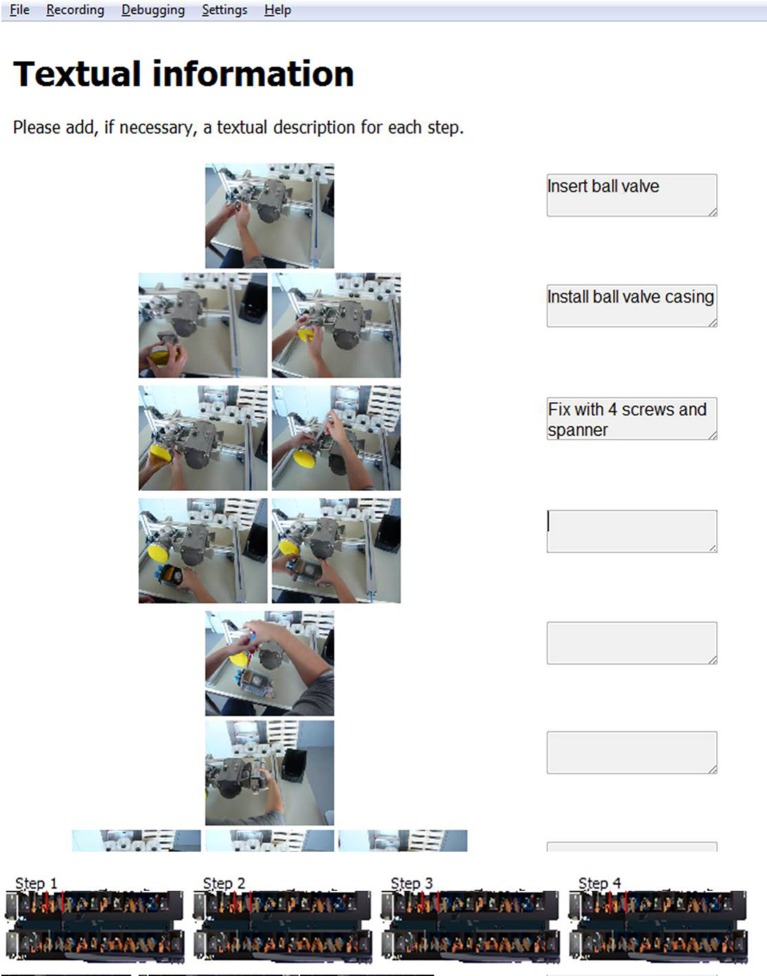
**Screenshot for step 3 within the IBES tool where users may edit textual description for each instructional step**.

In the third step within the IBES tool, subjects see their preliminary manual consisting of all instructional steps row by row along with their associated pictures (Figure 3). In this phase of the instruction design they can add textual descriptions for each step into the corresponding text box. In order to scroll up and down the users have to press the left mouse button while moving the mouse.

In step 4 within the IBES tool, the completed manuals are displayed and may be printed out. They are either ready for immediate use or users may manually add overlays, like arrows, boxes, circles, and so on.

In our experience, the entire four-step procedure takes around 12 min per every minute video. For our test cases (two videos with a total duration of about 5 min each) the average working time was 60 min.

Each of the described four steps within the IBES tool comes with a description for the users that informs them of what the step consists of and what they have to do (see the white box of Figure 1 and the tops of Figures 2, 3). The users of the IBES tool can change the texts according to their needs in the “.html” files (“Step1.htm,” “Step2.htm,” “Step3.htm,” “Step4.htm”) saved under the path “_global” → “PaperManual.” Additionally, within the “.html” files the users are free to replace or delete the given logo (“logo.png”).

### Output format

A folder is associated with each participant, identified by the subject ID. It consists of all “.html” files generated by the IBES workflow, so that all steps within the IBES tool can be tracked even after instruction creation. Furthermore, a “results.csv” file contains the subject ID, the fps, and for each segment the action ID, the start frame, the end frame, the text description, the number of chosen pictures, and the list of pictures. This file may be used for further statistical analyses in user studies and for event segmentation plots (please refer to the R code in the appendix).

## Validation of event segmentation based correlates of the IBES tool

We conducted two studies to validate the event segmentation based correlates of the IBES tool. In the first study, one group of participants designed instructions for two industrial scenarios by using the IBES tool (“instruction creation task”). In the second study (“event segmentation task”) participants segmented the videos of the same scenarios (Newtson and Engquist, 1976). We tested if the event boundaries resulting from the “instruction creation task” with the IBES tool are correlated to those in natural event perception assessed in the “event segmentation task.” The empirical question was if the participants' mental model of the task assessed during instruction creation using static frames of video is similar to the automatic event perception processes involved in online viewing of the video.

### Material

**Figure 4:**
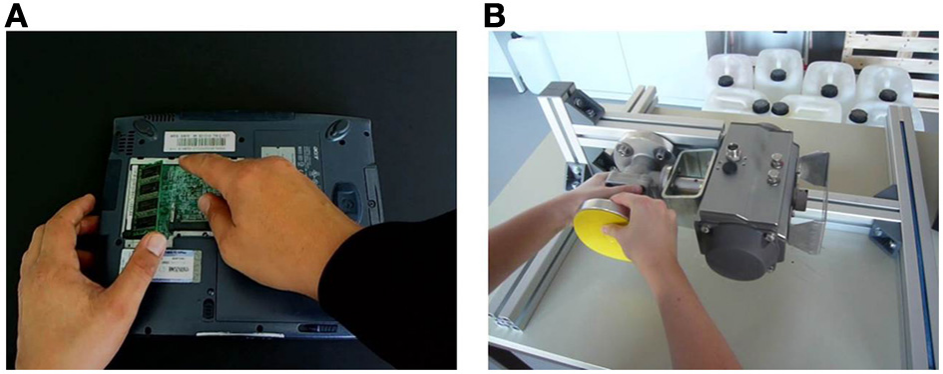
**Screenshots of (A) a maintenance task of installing a new notebook RAM and (B) an industrial manual task in which parts of a pump system are assembled**.

In both studies we used two industrial scenarios in which the actor performs some manual operations- one scenario involved changing a notebook RAM (similar to the video accessible from https://www.ict-cognito.org/demo.html)^1^ and the other scenario involved assembling a pump system (similar to the video accessible from https://www.ict-cognito.org/news.html)^2^. The videos of the tasks were recorded from a first-person perspective (Figure 4); the notebook scenario took 1 min and 12 s and the pump scenario took 3 min and 16 s.

### Participants

In the “instruction creation task” 20 participants (average age of *M* = 25.1 years, *SD* = 1.9) including 11 male and 9 female students from the University of Kaiserslautern created manuals for both scenarios with the IBES tool.

For the “event segmentation task” we recruited 22 new participants from the same university; 12 subjects segmented the video of the notebook task [6 females and 6 males with an average age of *M* = 25.5 years (*SD* = 1.9)] and 10 subjects segmented the video of the pump task [4 females and 6 males with an average age of *M* = 24.8 years (*SD* = 2.5)].

### Procedure

Here we review the study procedures of the “instruction creation task” and compare it with the “event segmentation task.” Participants in the “instruction creation task” saw a video of the notebook scenario first in order to become familiar with it. Then, they were introduced to the functionality of the IBES tool. They had to divide the whole scenario into steps that they thought will be “useful for giving instructions” by defining the start and end points of each instructional step, respectively. No time limit was given and participants had the opportunity to modify their choice of steps during segmentation. Afterwards, they sequentially assigned descriptive pictures and wrote textual explanations according to the sequence that they chose within the tool. Participants executed the same procedure a second time when they created instructions for the pump scenario.

During the “event segmentation task” participants saw the video in question three times; the first time without any instruction in order to get familiar with it and the second and third time to segment it into fine and coarse events while watching the videos. The order of fine and coarse segmentation was counterbalanced across participants. While watching the video they tapped a button whenever they thought one meaningful event ended and another meaningful event began (Newtson and Engquist, 1976).

To summarize, in the “instruction creation task” the identification of steps was offline, without any time constraints, and with the explicit aim to create instructions. In the “event segmentation task” the segmentation was “online.” The participants' task was to segment the video according to their subjective perception of fine and coarse event segments, respectively.

### Results

We analyzed participants' segmentation data with respect to the “instruction creation task” and the “event segmentation task.” We binned the data into 1 s intervals [adapted from Zacks et al. (2009)]. First, we present the data of the “event segmentation task.” Second, we describe the results of the “instruction creation task” and compare them to those of the “event segmentation task.”

#### Event segmentation task

**Figure 5:**
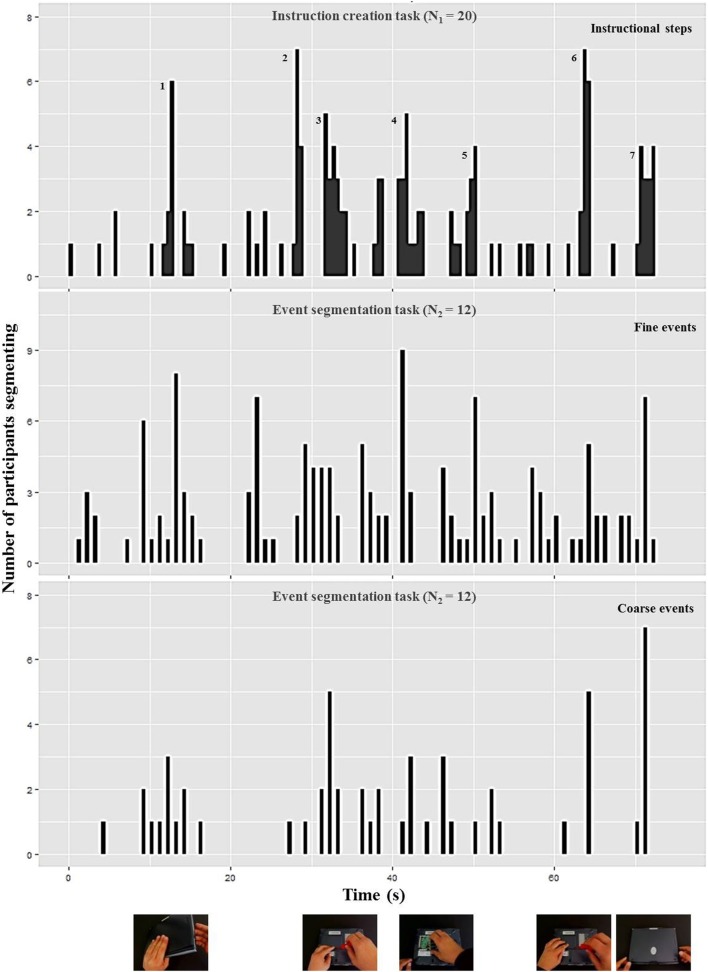
**Number of participants who identified the end point of a step (“instruction creation task,” upper diagram), a fine event boundary (“event segmentation task,” middle diagram), and a coarse event boundary (“event segmentation task,” lower diagram) during each 1-s interval of the notebook scenario [adapted from Zacks et al. (2009)].** The pictures below represent the notebook scenario at the corresponding coarse event boundary. The additional numbers in the upper diagram highlight high frequencies for defining ends of instructional steps corresponding to the mean and median numbers in Table 1 and the textual content in Table 2.

**Figure 6:**
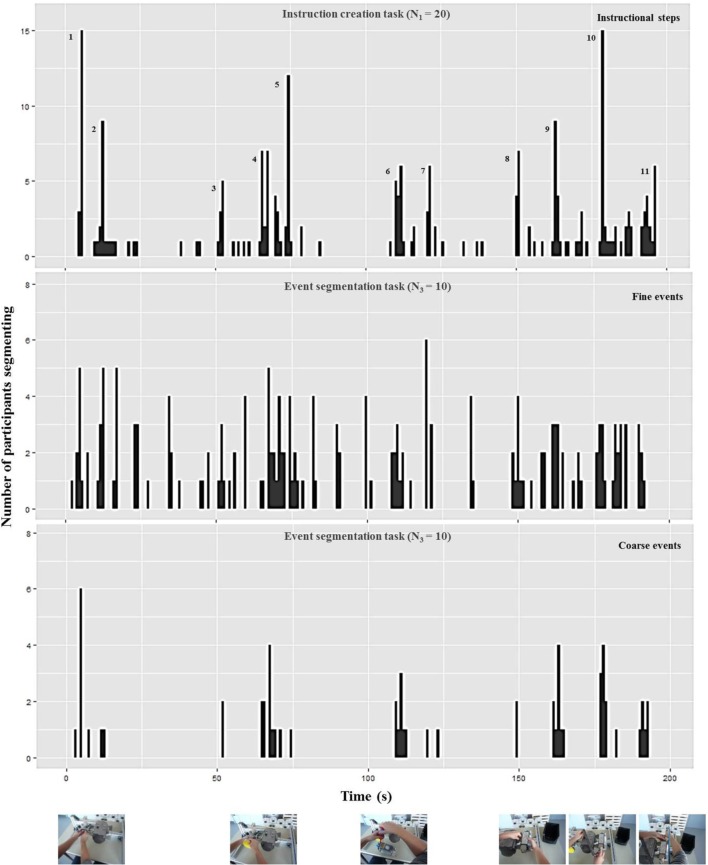
**Number of participants who identified the end point of a step (“instruction creation task,” upper diagram), a fine event boundary (“event segmentation task,” middle diagram), and a coarse event boundary (“event segmentation task,” lower diagram) during each 1-s interval of the pump scenario [adapted from Zacks et al. (2009)].** The pictures below represent the pump scenario at the corresponding coarse event boundary. The additional numbers in the upper diagram highlight high frequencies for defining ends of instructional steps corresponding to the mean and median numbers in Table 1 and the textual content in Table 2.

As evident in Figures 5, 6, participants perceived more event boundaries in the fine segmentation condition compared to the coarse segmentation. More specifically, participants created a mean number of 4.6 coarse event segments and 12.0 fine event segments for the notebook and 6.1 coarse event segments and 18.1 fine event segments for the pump scenario (see Table 1).

**Table 1:** **Number of steps during the “instruction creation task” and number of events in the coarse and fine condition during the “event segmentation task”**.

	***M***	**Median**	***SD***	**Min**	**Max**
**NOTEBOOK SCENARIO**					
**Instruction creation**					
Instructional steps	6.6	6.5	2.6	2	13
**Event segmentation**					
Coarse events	4.6	5.0	1.3	3	6
Fine events	12.0	11.5	4.5	5	19
**PUMP SCENARIO**					
**Instruction creation**					
Instructional steps	11.0	11.5	3.2	5	16
**Event segmentation**					
Coarse events	6.1	6.0	1.8	4	10
Fine events	18.1	18.5	6.7	10	26

For the coarse condition, there are five groups of event boundaries for the notebook scenario (Figure 5) and six groups of event boundaries for the pump scenario (Figure 6). All groups of event boundaries present in the coarse segmentation also occur in the fine-grained segmentation. Moreover, in the fine-grained condition there are additional event boundaries. For instance, in the pump scenario, some participants perceived the laying down of the spanner at the end of screwing as an additional segment, whereas in the coarse condition the majority of subjects grouped this action with the previous screwing. This suggests that participants perceive a hierarchically structured stream of information (Kurby and Zacks, 2007).

#### Instruction creation task

We analyzed the data of the instruction creation task by counting the end points of instructional steps identified by each participant (Newtson and Engquist, 1976). Results are plotted in Table 1. The median and mean numbers of instructional steps are 7 and 11 for the notebook and pump scenario, respectively (Table 1). These values go along with the numbers of observed groups of instructional steps displayed in the upper diagrams of Figures 5, 6 respectively.

In a second step, two independent raters analyzed the identified instructional steps across all participants including the textual descriptions and the points in time. One instructional step became part of a “consensus version” of the scenario in case both raters agreed that it was identified by at least half of all participants (*N* = 10). This resulted in 7 steps for the notebook and 11 steps for the pump scenario (see Table 2). In the upper diagrams of Figures 5, 6 we added those step numbers to the respective groups of event boundaries.

**Table 2:** **Consensus version for each scenario with steps being described during textual description by at least half (*N* = 10) out of all participants (*N* = 20)**.

**Notebook scenario**	**Pump scenario**
1. Turn the notebook upside down	1. Put ball valve into base
2. Unscrew both screws of the cover	2. Put casing onto base
3. Remove the cover	3. Fix with four screws
4. Insert the RAM	4. Tighten the screws with spanner
5. Put the cover on again	5. Put positioner covering on positioner
6. Screw both screws of the cover	6. Screw four screws
7. Turn the notebook back	7. Put the positioner onto the actuator
	8. Fix it with 2 nuts
	9. Tighten the nuts with spanner
	10. Connect actuator and positioner by pipe
	11. Connect the tube with the positioner

The mean and consensus number of instructional steps lie in between the number of events perceived during fine and coarse event segmentation. This indicates that the average structure chosen for instruction manual creation is a compromise between coarse and fine granularities. Furthermore, the comparison of the data shows that the structure of the manual is a combination of fine and coarse instructional steps. Each coarse event boundary found in the lower diagrams of Figures 5, 6 has a corresponding instructional step in the upper diagram; the remaining instructional steps that do not have a corresponding coarse event boundary can be found in the fine event segmentations shown in the middle diagrams.

The number of steps identified across participants in the “instruction creation task” ranged from 2 to 13 in the notebook and 5 to 16 in the pump scenario (Table 1). This result indicates that participants varied both toward more detailed and broader segmentations during instruction creation than the consensus versions listed in Table 2. Therefore, both raters analyzed all deviations from consensus (most frequently occurring boundaries) and agreed on 7 deviations for the notebook and 9 deviations for the pump scenario. In the following, we will describe a few examples of deviations in either direction compared to the consensus shown in Table 2.

On the one hand, there were participants who created more coarse instructional steps. In the manuals for the notebook scenario five participants summarized “Putting the cover on again” (see step 5 of the notebook scenario in Table 2) and “Screw both screws of the cover” (step 6) into one single step of “Closing the cover.” Similarly, steps 2 and 3 were summarized into “Open the cover.” A reduced notebook manual incorporating these consolidations would consist of five instructional steps. Consolidations for the pump scenario would result in a coarser pump manual of six steps according to the inspections of the raters. If participants segmented in a coarser way compared to consensus version of the pump scenario in Table 2, then they might merge steps 2 to 4, 5, and 6, and 7 to 9 into one step respectively. For instance, they did not segment “Put the positioner onto the actuator,” “Fix it with two nuts,” and “Tighten the nuts with spanner” (steps 7 to 9 from Table 2) into separate steps but perceived all three of them as one common step “Assemble the positioner onto the actuator.” The number of instructional steps in the coarser instruction manuals equals the mean number of coarse event boundaries (5 in the notebook and 6 in the pump scenario).

On the other hand, some manuals created by participants had a more detailed structure than indicated in Table 2. For instance, when the actor in the video screwed more than one screw in sequence, some participants across scenarios defined separate steps, e.g., “Screw first screw,” “Screw second screw,” and so on. A number of participants added instructional steps like “Initial state” and “Final state” to their manual. However, even the most detailed instruction manuals including 13 and 16 steps respectively (Table 1) did not reach the levels of fine granularity of the fine event segmentations (see 19 and 26 fine events in Table 1). For example, no subject understood laying down a tool as a separate instructional step whereas during fine event segmentation some participants did.

Finally, we analyzed if steps chosen in the “instruction creation task” were indeed based on complete natural event segments. In the analysis we correlated the number of steps chosen during the first step within IBES (i.e., the segmentation into instructional steps) and the number of images chosen during the subsequent step within IBES (i.e., the choice of appropriate pictures). If the participants selected fewer instructional step boundaries in the former and more number of frames in the latter to represent sub-events within the event, then we would expect a negative correlation. A negative correlation between the number of instructional steps and representative pictures within each step would indicate that the event boundaries were not complete but some relevant sub-steps are present within each event boundary. The average number of pictures per step was 2.3 with *SD* = 0.9 (in a range between 1.1 and 5.0 pictures per step) and no significant correlation was found between number of steps and pictures chosen per step (*r*_Pearson_ = −0.28, *p* = 0.078). Absence of a negative correlation indicates that the information content within an instructional step is not dependent on the number of chosen pictures per step.

Taken together, the results of this study show that the steps identified during instruction creation correspond to the event segmentation data. First, the graphical comparison shows an overlap between step boundaries from IBES segmentation and event boundaries from fine and coarse event segmentation. Second, we analyzed the instructional steps according to their points in time and content and found each of them being represented either as fine or coarse events. Thus, the proposed tool enables generation of user manuals that are based on the natural perception of dynamic activity.

## Discussion

We have described an easy-to-use, user-friendly tool for making instruction manuals from task videos. The aim of the paper was to demonstrate that the IBES tool helps users produce instruction manuals whose structure is based on the theory of natural event perception. Natural event perception yields event boundaries that play an important role in memory and learning (Newtson et al., 1977; Schwan and Garsoffky, 2004; Huff et al., 2012). For instructional material, literature indicates that structural design based on event boundaries enhances understanding and memory (Spanjers et al., 2010; Van Gog et al., 2010) whereas instructions that violate the existence of natural event boundaries decrease performance (Adamczyk and Bailey, 2004).

The empirical validation demonstrated that the steps created by using the IBES tool are correlated to event boundaries derived from event perception of dynamic activities. For creating a manual with the IBES tool, the user actively selects segments from a picture sequence derived from a task video by setting the beginning and end points of steps. The users may use multiple iterations for selecting, adding, and deleting steps for deciding on the best structure for the manual. We call this “offline event segmentation.” In contrast, in online event segmentation, the observer marks the end of one event and the beginning of the next event while passively watching an ongoing activity using the established method of button presses while watching a video (Newtson and Engquist, 1976; Zacks et al., 2001). We compared the resulting boundaries from offline and online event segmentation for two scenarios. Even if the segmentation task within the IBES tool is different from the established procedure of recording online event boundaries, it yields steps that correlate with event boundaries identified in online event segmentation. This correlation holds independent of the scenario.

A comparison between the results from the instruction creation task and the event segmentation task shows that each instructional step within the IBES tool has a corresponding event boundary resulting from coarse and fine event segmentation. We showed that, similar to event boundaries in event segmentation, also step boundaries assessed with the help of the tool are strategic points, i.e., summaries of the preceding activities into one conclusive step (Kurby and Zacks, 2007; Schwan and Garsoffky, 2008). This is supported by the fact that no negative correlation between number of steps and number of chosen pictures per step was found. Pictures within one step do not reflect more information on relevant sub-steps; they rather seem to serve as an additional description of the activity.

All event boundaries of the coarse events appear in the segmentation of instructional steps; a reduced number of instructional steps chosen for the manual are comparable to the number of coarse event boundaries. In contrast, the maximum number of instructional steps does not reach the maximum number of fine events from online event segmentation. The finer segments result from very small changes in movement (Zacks et al., 2009) but these may be irrelevant for giving instructions about the task. For instance, each picking up and putting down of tools can be perceived as new events but are not important as instructional steps. The mean number of instructional steps was in between the means for coarse and fine segments from online event segmentation. This suggests that instruction creation without any explicit instructions for granularity of events results in a compromise between detailed and still clear information. Manuals may be indeed very detailed for some subjects but still not as fine-grained as in the fine segmentation. Some of the finer segments of online event segmentation probably get combined into a single step in offline manual creation.

Similar to variability in natural event segmentation, we found that participants differ in segmentation of steps during instruction creation. This observation goes along with findings from event segmentation (e.g., Massad et al., 1979) where, for instance, higher expertise level influenced the number of event boundaries (Graziano et al., 1998, for an overview see Schwan and Garsoffky, 2008). Graziano et al. (1998) investigated different instructions, e.g., to learn the presented content, which resulted in fewer event boundaries. Furthermore, when provided with relevant information prior to the task, children segmented the ongoing behavior into larger events than novices. Dividing on a higher-level, i.e., segmenting into fewer event boundaries might reflect greater expertise in subjects. The users may use the IBES tool to divide the video according to their perception of event boundaries that is correlated with their level of expertise or familiarity of the task.

The variability in granularity during instruction creation is useful. The deviations offer the possibility to create more detailed or rather coarse instructions depending on the need for support of a user. Creating these different versions of manuals may meet the needs of different user groups that require assistance for the same task. Whereas more experienced users may prefer communication of only main steps, novices may prefer more information on how to accomplish the steps (Massad et al., 1979; Eiriksdottir and Catrambone, 2011). Deviations across manuals are furthermore useful in order to define a hierarchical structure of instructions with main steps derived from a version where a user defined broad steps and subordinate steps derived from a user defining more detailed steps. Previous work confirmed the usefulness of such a hierarchical structure as a design principle for procedural instructions (Zacks and Tversky, 2003).

### Practical implications

We presented a tool with which a person can conveniently design instructions for a task only based on its video. While current approaches indicate that naïve users are capable of instruction creation (Agrawala et al., 2003; Tversky et al., 2006; Daniel and Tversky, 2012), we present the first software tool that conveniently enables them to do it. A manual produced by help of a simple user interface contains a combination of pictorial explanations and textual descriptions as well as potential visual cues that can be added manually stressing important or crucial operations for each step. So, the tool provides informally and immediately well-structured and well-designed support of assembly workers (Mayer et al., 2005; Horz and Schnotz, 2008) with little effort of only one person using the tool.

The IBES software tool opens up new avenues for empirical investigations of instructional material with respect to placement of pauses, structure of steps, and individualization of manuals depending on expertise (Zacks and Tversky, 2003; Eiriksdottir and Catrambone, 2011; Spanjers et al., 2012). For instance, manuals resulting from IBES could be experimentally tested against manuals violating the existence of event boundaries. In addition, the event boundaries assessed by help of the tool may be the basis for general and more formal design decisions. For instance, when incorporating individual differences in granularity of segmentation, the tool offers two different use cases. On the one hand, a novice user gets a detailed step-by-step manual; on the other hand, experienced users get a higher-level manual that acts as a commemorative support for them. A further option is to combine higher-level and lower-level steps in order to provide a hierarchically structured manual containing main and relevant subordinate steps.

Finally, the tool enables a convenient communication between the users of instructions and the designers of instructions or instructional researchers. Input from users' design may be used across different tasks, e.g., varying difficulty (Zäh et al., 2009) or different user groups, e.g., varying expertise (Eiriksdottir and Catrambone, 2011; Woestenenk, 2011) which in a further step enables the tailoring of instructions to the target group or even find empirically derived design principles that are specific enough for the kind of task in question (Zwaga et al., 1999; Martin and Smith-Jackson, 2005).

### Comparison to other means to create instruction manuals

There is very little software support targeted to instruction manual generation. Engineers and trainers typically use existing data from the engineering process, e.g., graphical product models and planned production sequence data from CAD software like AutoCAD and import this information to word processing or image editing programs, e.g., MS Office Word and PowerPoint, for additional, manually edited descriptions and graphics. Technical writers and editors use these documents as a starting point, and may exploit more sophisticated and expensive desktop publishing tools, such as, Adobe InDesign for more powerful functionalities for graphic design and media creation. However, this process and the software tools above are not targeted toward instruction creation. Thus, instruction manual creation is labor-intensive, *ad-hoc*, with a steep learning curve, and involves expensive iterations when the manual does not match its purpose. Furthermore, the process does not produce additional artifacts such as log files with which researchers may further investigate the design process.

The IBES tool addresses these issues by featuring instruction creation based on cognitive science principles, an easy-to-use user interface, log files immediately available for use for further statistical analyses (see the R commands in the appendix on the last page of this paper). It is freely accessible, as opposed to commercial software tools with high license fees. With the help of IBES, instructional designers in professional and scientific settings will be able to create multimedia manuals which otherwise would require numerous cumbersome steps with multiple different software tools (e.g., tools for video, screenshot, word, and image processing).

### Future directions

The support of instruction creation is being actively investigated in the area of artificial intelligence where methods of, e.g., computer vision (Chellappa et al., 2008) and advanced motion tracking (Petersen and Stricker, 2009; Bleser et al., 2011), are supposed to make sense of an ongoing human activity and (semi-)automate the process of instruction creation (Tversky et al., 2006; Worgan et al., 2011; Petersen and Stricker, 2012). Some of these approaches automatically segment activities and provide instructions for them. Since one of their requirements is to meet the actual human understanding of the activity within the manual (Petersen and Stricker, 2012), the IBES tool offers an approach to investigate how people understand and elaborate information of ongoing activities.

## Conclusion

Producing efficient instruction manuals currently requires an effortful, labor intensive process involving creation of meaningful structure for the assembly steps and the choice of appropriate media. We presented a tool with which users can easily generate a multimedia manual based on only a video of the assembly task in question. In an empirical validation we showed that the resulting manuals conform to event boundaries perceived during event perception. IBES incorporates both organization by event boundaries, which facilitates understanding and memory, and multimedia instructions, which facilitate learning and understanding. As our society creates more and more complex artifacts, IBES is an important means to convert procedural information into effective procedural instructions.

### Conflict of interest statement

The authors declare that the research was conducted in the absence of any commercial or financial relationships that could be construed as a potential conflict of interest.

## Footnotes

^1^Please scroll down to “Augmented Reality Handbook (December 2012, DFKI).”

^2^Please scroll down to “10-13.09.2012—New challenging workflow captured in SmartFactory.”

## Appendix

### Statistical analyses and event segmentation plot with R

#- Put script file and output files in the same directory

#- get working directory by using setwd() or getwd()

wd <- getwd()

#- Import the data “results_1.csv”,…

paths <- dir(path = wd, pattern = “^results_”, full = T)

dat.raw <- lapply(paths, read.csv)

#- Generate data frame

dat <- data.frame(dat.raw[1])

for (i in 2:length(dat.raw))

{

dat <- rbind(dat,data.frame(dat.raw[i]))

}

#- Plot the segmentation structure unsing qplot() of the ggplot2 package

#- bin width set to 1000 ms; can be adjusted by modifiying the x parameter in qplot()

library(ggplot2)

qplot(stop/fps,data=dat,binwidth=1,xlab=“Time (s)”,ylab=“Number of Partcipants Segmenting”)
